# A Cross-Matrix Peripheral Transcriptional Signature in Suicide Attempt: *NR3C2* Downregulation in Blood and Buccal Swab Samples

**DOI:** 10.3390/ijms27167185

**Published:** 2026-08-11

**Authors:** Zeynep Ozan, Goksu Kasarci-Kavsara, Saim Pamuk, Oguz Polat, Sinem Bireller

**Affiliations:** 1 Department of Forensic Sciences, Graduate School of Health Sciences, Acibadem Mehmet Ali Aydinlar University, 34752 Istanbul, Türkiye; 2 Department of Molecular Medicine, Aziz Sancar Institute of Experimental Medicine, Istanbul University, 34093 Istanbul, Türkiye; 3 Department of Otorhinolaryngology & Head and Neck Surgery, Bakırköy Dr. Sadi Konuk Training and Research Hospital, 34147 Istanbul, Türkiye; 4 Department of Forensic Medicine, School of Medicine, Acibadem Mehmet Ali Aydinlar University, 34752 Istanbul, Türkiye; 5 Department of Biochemistry, Faculty of Pharmacy, Acibadem Mehmet Ali Aydinlar University, 34752 Istanbul, Türkiye

**Keywords:** suicide attempt, *NR3C2*, HPA axis, peripheral gene expression, buccal swab

## Abstract

The biological basis of suicidal behavior extends beyond psychiatric diagnosis, and alterations in stress-response regulation, monoaminergic signaling, and GABAergic function may contribute to suicide risk. We enrolled 69 adults presenting to the emergency department after a suicide attempt episode and 72 controls. Expression of eight candidate genes (*SLC6A3*, *SLC6A4*, *NR3C1*, *NR3C2*, *DRD2*, *MAOA*, *GABRA2*, and *GABBR2*) was measured by qRT-PCR in paired peripheral blood and buccal swab samples, with *GAPDH* as the reference gene. Multiple testing was addressed with the Benjamini—Hochberg false discovery rate. *NR3C2*, which encodes the mineralocorticoid receptor, was the only gene downregulated in both sample types after FDR correction (blood: q = 0.0007; swab: q = 0.0004). Blood additionally showed changes in *SLC6A4* (↓), *DRD2* (↑), and *GABBR2* (↑). In buccal swabs, every gene that reached significance—*SLC6A3*, *NR3C1*, *GABRA2*, and *GABBR2*—was downregulated. No gene achieved AUC ≥ 0.90 in ROC analysis. Gene-by-biochemistry correlations did not survive FDR correction, suggesting transcriptional changes independent of acute metabolic status. Overall, peripheral expression was matrix-dependent, and *NR3C2* downregulation stood out as the one signal that replicated across both. Blood and buccal swabs captured complementary biological information, together pointing to impaired HPA-axis buffering. Because group-level confounders could not be adjusted for, these findings should be read as hypothesis-generating and warrant confounder-controlled, longitudinal validation.

## 1. Introduction

Suicide attempt cannot be explained by psychiatric diagnosis alone. In clinical practice, patients with similar diagnoses may have very different levels of suicidal risk. This indicates that diagnosis, although clinically necessary, does not fully capture the vulnerability that may lead from psychiatric distress to suicidal behavior. Risk assessment tools and psychometric scales are useful in routine evaluation, but they remain limited in explaining why suicidal behavior develops in some patients and not in others under apparently similar clinical conditions. The stress–diathesis model addresses this problem by separating relatively stable vulnerability factors from more immediate clinical or environmental triggers [1,2]. What the model proposes is not that stress causes suicide, but that stress acts on a biologically susceptible substrate—and it is this substrate that determines whether a clinical threshold is crossed. This study focuses on three of them: HPA-axis regulation, monoaminergic signaling, and GABAergic inhibitory control—systems that intersect rather than operate in parallel [1,2,3].

Serotonergic dysfunction has been widely discussed in relation to suicidal behavior. It has been associated with impulsivity, aggression, mood dysregulation, and suicidal acts, although findings differ across study populations and methods [1,4]. *SLC6A4* encodes the presynaptic serotonin transporter and has therefore been frequently examined in this field. Previous genetic studies have reported associations between *SLC6A4* variants and suicidal behavior, but these associations have not been consistent across cohorts [5]. One reason for this inconsistency may be that genetic variation reflects inherited susceptibility, but not necessarily the biological state of the individual at the time of clinical presentation. Gene expression studies may therefore add a different layer of information, especially when suicidal behavior is examined during an acute clinical encounter. In this context, altered *SLC6A4* mRNA expression in peripheral blood has been linked with worsening suicidal ideation and suicide attempts during follow-up of major depressive episodes [6].

Serotonergic regulation should also be considered together with other monoaminergic pathways. *SLC6A3* encodes the dopamine transporter, which regulates presynaptic dopamine reuptake and may also influence serotonergic tone through cross-regulatory interactions between dopaminergic and serotonergic systems [7]. *MAOA* produces an enzyme that breaks down serotonin, dopamine, and related monoamines. Reduced activity has been tied to impulsivity and suicidal behavior, with the association appearing most clearly in individuals who experienced early adversity [8,9]. Taken across these three genes, monoaminergic involvement in suicidal behavior is not a single-system story—it runs across pathways that are biologically entangled.

Dopaminergic signaling is relevant to behavioral dimensions that frequently intersect with suicidal behavior, including reward processing, motivation, impulsivity, and risk-taking. *DRD2* encodes the dopamine D2 receptor, one of the main receptors involved in dopaminergic neurotransmission. Disturbances in *DRD2*-related signaling may therefore be important particularly in individuals in whom suicidal behavior overlaps with impulsive traits, substance use, or impaired behavioral inhibition [10]. This receptor-level relevance is reinforced by genetic evidence: the largest multi-ancestry GWAS meta-analysis of suicide attempt to date identified *DRD2* among its genome-wide significant loci, independently supporting its inclusion as a candidate gene in this panel [11]. Genetic and transcriptomic studies also indicate that dopaminergic and serotonergic genes do not act as isolated systems, but may contribute to shared behavioral phenotypes, including impulsivity, externalizing traits, and substance use [5,12]. Still, peripheral mRNA data should be interpreted carefully. Transcript levels do not always correspond to receptor or transporter activity at the protein level, especially in dopaminergic pathways [13]. For this reason, peripheral expression data should be used cautiously. In the present study, these data are interpreted as peripheral transcriptional findings, not as direct evidence of central dopaminergic function.

Stress-related regulation was also central to the gene panel used in this study. The HPA axis links stress exposure with endocrine adaptation and neurotransmitter-related processes. Cortisol acts mainly through two intracellular receptors: the glucocorticoid receptor, encoded by *NR3C1*, and the mineralocorticoid receptor, encoded by *NR3C2*. Because of its high affinity for cortisol, *NR3C2* is more closely related to basal HPA-axis tone and recovery after stress. *NR3C1*, in contrast, has a stronger role in feedback regulation during stress responses [14]. Previous studies have associated altered *NR3C1* and *NR3C2* expression with suicide attempt risk, particularly in the context of childhood trauma. Lower *NR3C2* expression has been linked to trauma burden, whereas *NR3C1* expression appears to vary according to trauma subtype and clinical context [14]. Therefore, both receptors are relevant for evaluating stress-related transcriptional changes in suicidal behavior. *NR3C2* itself has not emerged as a genome-wide significant locus in large suicide attempt GWAS, unlike *DRD2*. That absence is not unusual for a gene whose contribution to risk works through gene-by-environment mechanisms rather than through common inherited variation. This is consistent with the trauma-linked expression pattern already discussed above [14], and with genome-wide methylation studies of suicide that have found extensive epigenetic dysregulation across stress-response pathways, distinct from the additive genetic effects GWAS is built to detect [15]. A gene acting mainly through environmentally responsive regulation can produce a real, reproducible expression signal without leaving a trace in a common-variant association study, which is why candidate-gene expression findings and GWAS results are not expected to align for every HPA-axis target.

The HPA axis is also difficult to separate from monoaminergic signaling. Long-term stress and altered cortisol regulation may affect serotonergic receptor sensitivity and serotonin synthesis. At the same time, serotonergic dysfunction may increase the reactivity of the HPA axis to stressors [16,17]. This crosstalk between the HPA axis and monoaminergic pathways is one reason the gene panel in this study spans both systems. Suicidal behavior is unlikely to have a single neurotransmitter explanation, and studying these genes in isolation from each other would miss that. The data from both systems are better read together than apart.

The third component of this panel concerns GABAergic inhibitory signaling. Prefrontal and cortico-limbic circuits depend on GABAergic interneurons to regulate stress responses, emotional processing, and behavioral inhibition—functions that are clearly relevant to suicidal behavior. Postmortem studies of individuals who died by suicide have found epigenetic silencing of GABAergic genes in brain tissue, pointing to a disruption in inhibitory regulation that may be mechanistically important [15,18]. Chronic stress and prolonged glucocorticoid exposure may also influence GABAergic receptor expression, linking inhibitory signaling to HPA-axis regulation [19]. In this study, this pathway was represented by *GABRA2* and *GABBR2*. *GABRA2* encodes the α2 subunit of the GABA-A receptor, whereas *GABBR2* encodes the GABA-B receptor.

What molecular studies have added to suicide research is substantial. What they have not solved is the translation problem. A large part of the current evidence comes from postmortem brain studies, genetic association studies, and large-scale omics analyses. Each of these approaches is informative, but their scope is different from a peripheral expression study conducted in living patients. Postmortem brain tissue can provide mechanistic information, yet it cannot reflect the biological status of patients at the time of emergency department admission. Genetic association studies are also useful for identifying susceptibility loci, but they do not show whether a gene is actively up- or downregulated in a specific tissue or clinical state [11,20]. More recent transcriptomic, epigenomic, and metabolomic studies have further shown that suicidal behavior is associated with broad molecular changes involving neurotransmission, inflammatory signaling, stress-related pathways, and synaptic regulation [15,21,22]. The gap between what is known at the molecular level and what can be measured at the bedside remains wide.

Peripheral gene expression analysis may help address part of this gap, particularly in emergency department settings where rapid and minimally invasive sampling is needed. At the same time, peripheral tissues should not be treated as direct substitutes for brain tissue. Gene expression patterns differ across tissues, and peripheral samples may reflect systemic biological responses, immune-endocrine interactions, acute stress physiology, medication effects, or tissue-specific regulation rather than central nervous system activity alone [22,23]. This does not weaken the value of peripheral expression data; it defines the level at which such data should be interpreted. Peripheral findings may provide clinically accessible information related to suicidal behavior if they are not presented as a direct molecular mirror of the brain.

Peripheral blood is one of the most used matrices in psychiatric biomarker research because it is accessible and biologically informative. Blood-based expression profiles may capture immune-endocrine interactions and acute systemic responses, both of which are relevant in patients presenting after a suicide attempt. However, blood gene expression may also be affected by acute stress, metabolic status, inflammation, medication use, substance exposure, and the physiological consequences of the attempt itself. Buccal epithelial samples represent a different peripheral compartment. They are non-invasive, easy to collect, and practical in emergency or forensic settings. RNA-based approaches in blood, saliva, and buccal-related samples also support the feasibility of expression analysis in accessible biological materials [24,25]. Because blood and buccal swabs differ in cellular composition and biological responsiveness, their paired evaluation may help distinguish findings that are shared across peripheral compartments from those that are specific to a given matrix.

In suicidal behavior and self-harm specifically, both systemic stress physiology and tissue-specific regulatory processes may leave measurable biological traces—and these traces need not be identical across sample types. Relying on a single peripheral matrix therefore risks missing part of the picture. Collecting blood and buccal swab samples from the same individuals at the same time point makes it possible to ask directly whether any given expression change is a shared peripheral signal or one that depends on the sampling matrix examined. A similar direction of change in both matrices may indicate a more stable peripheral pattern. In contrast, matrix-specific changes may suggest that blood and buccal epithelial samples provide complementary, rather than interchangeable, biological information. Buccal epithelial cells are of neuroectodermal origin and share a closer embryonic lineage with neural tissue than do blood-derived cells of mesodermal origin. This biological distinction provides a rationale for expecting that buccal and blood samples may yield partially non-overlapping transcriptional information and supports their joint evaluation rather than treating one as a simple substitute for the other [24].

To our knowledge, no previous study has comparatively examined the expression of multiple genes previously linked to stress-axis function, monoaminergic tone, and inhibitory signaling in paired peripheral blood and buccal swab samples collected from the same individuals presenting after a suicide attempt. In the present study, we analyzed the expression levels of *SLC6A3*, *SLC6A4*, *DRD2*, *MAOA*, *NR3C1*, *NR3C2*, *GABRA2*, and *GABBR2* in both peripheral blood and buccal swab samples from adults presenting to the emergency department due to a suicide attempt and from control individuals presenting for other reasons. We also evaluated whether gene expression findings were associated with selected routine biochemical parameters, including markers of metabolic and electrolyte status, to explore possible systemic correlates. By comparing two clinically accessible peripheral matrices, this study aimed to determine whether a suicide attempt is associated with a consistent or matrix-dependent peripheral transcriptional pattern involving the three regulatory systems this panel was designed to interrogate. A hypothesis-driven, candidate-gene design was used rather than an untargeted transcriptome-wide approach: the three systems examined here already have converging support from postmortem, genetic, and peripheral-expression literature, and a targeted panel preserved adequate statistical power within a sample size feasible for recruitment in an acute emergency-department setting.

## 2. Results

Gene expression levels of eight target genes were compared by qRT-PCR in paired peripheral blood and buccal swab samples collected from the case group (suicide attempt; *n* = 69) and the control group (*n* = 72).

### 2.1. Sociodemographic and Clinical Characteristics of Cases and Controls

A total of 141 adults were enrolled in the study (Table 1): 69 individuals who presented to the emergency department following a suicide attempt and 72 controls who presented for other reasons. The two groups were comparable with respect to age (*p* = 0.439), marital status, and place of birth. Sex distribution differed significantly between groups (*p* = 0.003): 71.0% of cases were female, and 27.5% were male, compared with 43.1% female and 55.6% male in the control group.

Regarding clinical characteristics, the case group showed markedly higher rates of psychiatric diagnosis history (59.4% vs. 1.4%; *p* < 0.001) and reported psychiatric medication use (*p* < 0.001). Substance and alcohol use was identified exclusively in the case group (*p* = 0.019). Among cases, 63.8% were presenting following their first attempt; 15.9% had made a second attempt, 8.7% a third, and 2.9% a fourth (8.7% unknown). Drug or substance ingestion was the predominant method of attempt (91.3%); all higher-lethality methods—including hanging, jumping from height, and use of sharp or cutting instruments—were observed exclusively in male individuals. The sex imbalance between groups and the restriction of substance use to cases were noted as potential confounding factors in the interpretation of subsequent findings.

### 2.2. Blood-Based Gene Expression Profiles

Four genes showed statistically significant between-group differences in blood samples that remained significant after FDR correction (Table 2).

**Figure 1:**
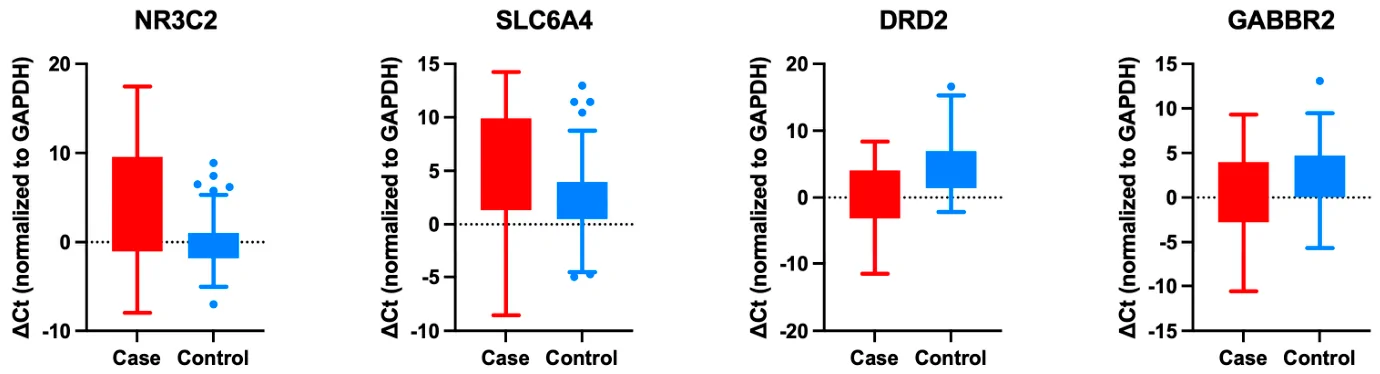
Peripheral blood ΔCt expression of FDR-significant genes in suicide attempt cases versus controls. Boxes indicate the 25th–75th percentile range with the median line; whiskers represent Tukey’s 1.5 × IQR criterion; individual points beyond whiskers are shown. Red: cases; blue: controls. q values (Benjamini–Hochberg FDR correction across eight genes): *NR3C2*, q = 0.0007; *SLC6A4*, q = 0.0007; *DRD2*, q = 0.0001; *GABBR2*, q = 0.0317. Sample sizes: *NR3C2*—cases *n* = 57, controls *n* = 63; *SLC6A4*—cases *n* = 62, controls *n* = 67; *DRD2*—cases *n* = 64, controls *n* = 63; *GABBR2*—cases *n* = 65, controls *n* = 65. ΔCt values are normalized to *GAPDH*; higher ΔCt corresponds to lower expression.

*NR3C2* expression was markedly downregulated in the case group (Figure 1) (log_2_FC = −4.59; q = 0.0007; Hedges g = +0.82). *NR3C2* encodes the mineralocorticoid receptor (MR), which binds cortisol with approximately tenfold higher affinity than the glucocorticoid receptor and plays a primary role in regulating basal HPA-axis tone and post-stress recovery. The downregulation observed in blood may therefore reflect a measurable peripheral correlate of impaired stress-buffering capacity [14,17,18]. In the same direction, *SLC6A4*, which encodes the serotonin transporter, was also reduced in cases (log_2_FC = −2.97; q = 0.0007; Hedges g = +0.60). Peripheral *SLC6A4* mRNA has previously been associated with the longitudinal course of suicidal ideation, and the downregulation observed here is consistent with the view that peripheral expression may capture meaningful aspects of the acute biological state [6]. By contrast, *DRD2*, encoding the dopamine D2 receptor, showed higher expression in the case group (log_2_FC = +2.54; q = 0.0001; Hedges g = −0.98), and had the largest absolute effect size among all eight genes. This result sits in tension with the dopaminergic hypofunction typically linked to suicidal behavior in the literature; as peripheral mRNA levels may not directly reflect central receptor function, this result should be interpreted with appropriate caution [10,13]. *GABBR2* also showed a modest but significant increase in blood (log_2_FC = +0.90; q = 0.0317; Hedges g = −0.56). Notably, as detailed in the cross-matrix analysis below, *GABBR2* moved in opposite directions across matrices—upregulated in blood but downregulated in buccal swabs. No significant differences were found for *SLC6A3*, *NR3C1*, *MAOA*, or *GABRA2* in blood (all q > 0.12).

### 2.3. Gene Expression Profiles in Buccal Swab Samples

**Figure 2:**
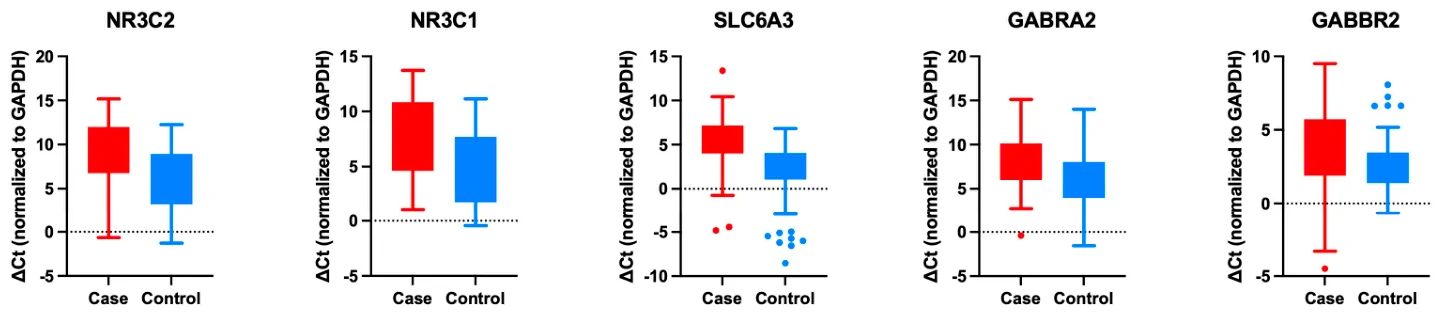
Buccal swab ΔCt expression of FDR-significant genes in suicide attempt cases versus controls. Boxes indicate the 25th–75th percentile range with the median line; whiskers represent Tukey’s 1.5 × IQR criterion; individual points beyond whiskers are shown. Red: cases; blue: controls. All genes reaching FDR significance in buccal swabs showed a consistent direction of downregulation in cases. q values (Benjamini–Hochberg FDR correction across eight genes): *NR3C2*, q = 0.0004; *NR3C1*, q = 0.0077; *SLC6A3*, q < 0.0001; *GABRA2*, q = 0.0077; *GABBR2*, q = 0.0077. Sample sizes: *NR3C2*—cases *n* = 31, controls *n* = 54; *NR3C1*—cases *n* = 19, controls *n* = 47; *SLC6A3*—cases *n* = 34, controls *n* = 64; *GABRA2*—cases *n* = 29, controls *n* = 51; *GABBR2*—cases *n* = 28, controls *n* = 63. The *NR3C1* comparison rests on 19 case samples; the confidence interval should be interpreted accordingly. ΔCt values are normalized to *GAPDH*; higher ΔCt corresponds to lower expression.

Sample sizes varied across genes in buccal swabs, reflecting differential RNA yield and quality among samples of this matrix (Section 4.4); genes are therefore reported with their respective *n* for cases and controls throughout. A greater number of genes showed significant between-group differences in buccal swab samples compared with blood, and all these differences pointed in the same direction: downregulation in the case group. Expression of *NR3C2* (log_2_FC = −4.91; q = 0.0004; Hedges g = +0.94), *NR3C1* (log_2_FC = −2.69; q = 0.0077; Hedges g = +0.86), *SLC6A3* (log_2_FC = −2.54; q < 0.0001; Hedges g = +0.93), *GABRA2* (log_2_FC = −2.74; q = 0.0077; Hedges g = +0.69), and *GABBR2* (log_2_FC = −1.82; q = 0.0077; Hedges g = +0.51) was significantly reduced in cases (Table 3, Figure 2). The *MAOA* and *NR3C1* comparisons rested on the smallest case samples in this matrix (*n* = 11 and *n* = 19, respectively); the corresponding effect sizes and confidence intervals should be read accordingly.

The consistent downregulation across all significant genes in buccal swabs is directionally aligned with the pattern of GABAergic gene silencing previously reported in the prefrontal cortex of individuals who died by suicide [15,18], suggesting that suppression of inhibitory control and glucocorticoid feedback mechanisms may be detectable at the peripheral level. *MAOA* showed the largest effect size among all swab findings (log_2_FC = −5.10; Hedges g = +1.26; q = 0.0030); however, this comparison rested on only 11 case samples, and the correspondingly wide confidence interval means that this result should be treated with caution. Neither *SLC6A4* nor *DRD2* reached significance in buccal swabs.

### 2.4. Matrix-Dependent Expression Pattern Across Blood and Buccal Swabs

**Figure 3:**
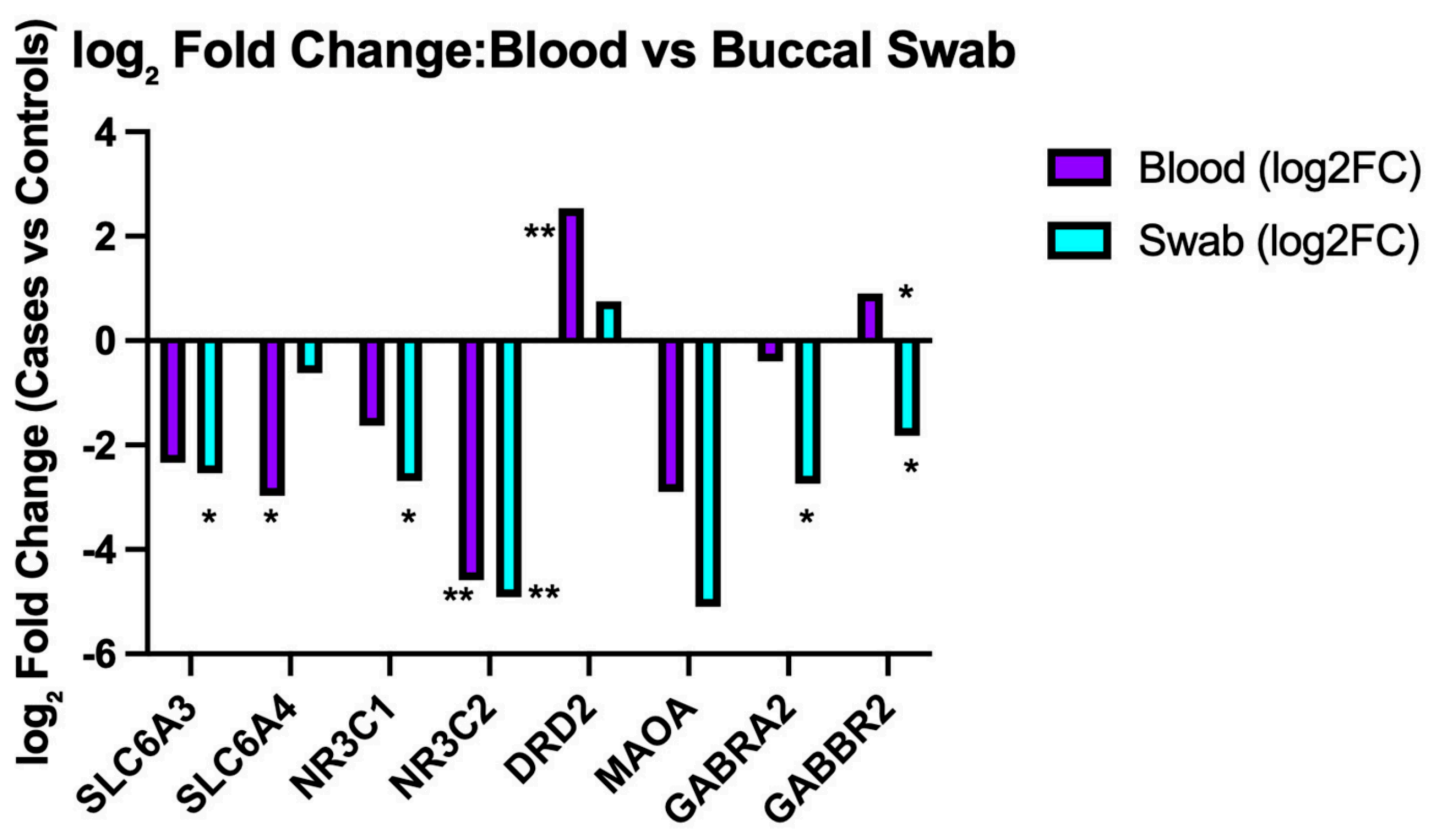
log_2_ fold change (log_2_FC) of eight target genes in peripheral blood and buccal swab samples from suicide attempt cases versus controls. Positive values indicate higher expression in cases; negative values indicate lower expression in cases. Purple bars: blood; cyan bars: buccal swab. Asterisks denote FDR-corrected significance (q < 0.05, Benjamini–Hochberg correction across eight genes per matrix). Exact q values—Blood: *SLC6A4*, q = 0.0007; *NR3C2*, q = 0.0007; *DRD2*, q = 0.0001; *GABBR2* q = 0.0317. Buccal swab: *SLC6A3*, q < 0.0001; *NR3C1*, q = 0.0077; *NR3C2*, q = 0.0004; *MAOA*, q = 0.0030; *GABRA2*, q = 0.0077; *GABBR2*, q = 0.0077. *NR3C2* was the only gene reaching significance in both matrices and in the same direction. *GABBR2* showed opposite directions across matrices (upregulated in blood, downregulated in buccalswab). The *MAOA* buccal swab result is based on 11 case samples and should be interpreted with caution. (* <0.05, ** <0.01).

When the two sample types were evaluated side by side, most genes showed a matrix-specific expression profile, with only one gene—*NR3C2*—showing consistent downregulation across both biological compartments (Figure 3). *SLC6A4* and *DRD2* reached significance exclusively in blood, whereas *SLC6A3*, *NR3C1*, *MAOA*, and *GABRA2* did so only in buccal swabs (Table 4). Blood and buccal epithelium are not measuring the same thing.

Buccal epithelium and blood differ in both cellular composition and developmental origin. This difference may partly explain why several receptor-related and GABAergic genes showed greater changes in buccal swabs than in blood. Across the dataset, *NR3C2* was the only gene downregulated in both matrices, with FDR-significant changes in blood (q = 0.0007) and buccal swabs (q = 0.0004). No other gene replicated across compartments. *GABBR2* stood out for a different reason: it moved in opposite directions—up in blood, down in swabs. Two matrices, same gene, opposite signals. That alone is enough to rule out treating them as interchangeable.

### 2.5. Discriminative Performance of Individual Genes

**Figure 4:**
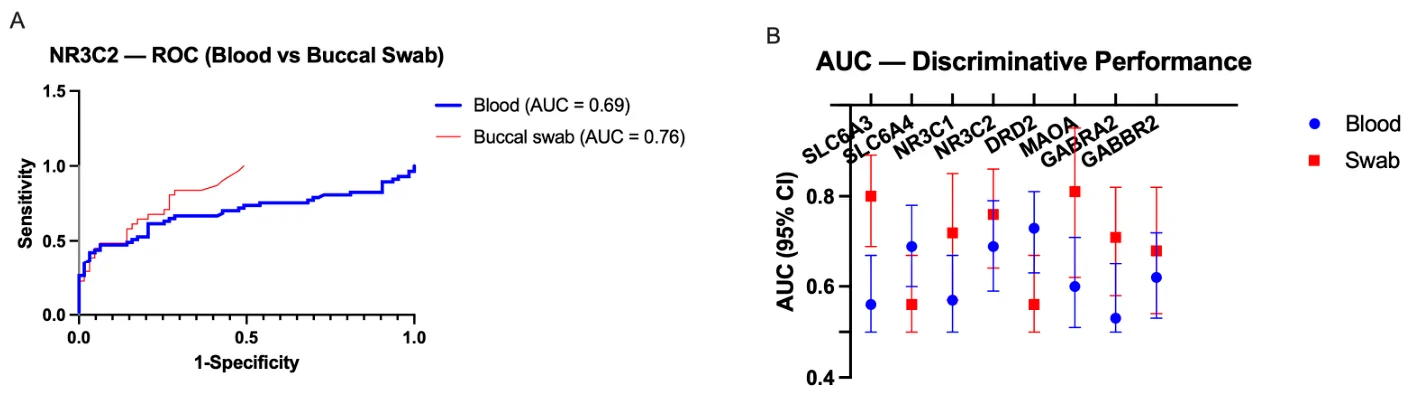
Discriminative performance of *NR3C2* and all target genes across peripheral blood and buccal swab samples. (**A**) Receiver operating characteristic (ROC) curves for *NR3C2* ΔCt expression in peripheral blood (blue) and buccal swab (red) samples. Area under the curve (AUC) values were estimated by bootstrap resampling (1000 iterations): blood, AUC = 0.69 (95% CI 0.59–0.79); buccal swab, AUC = 0.76 (95% CI 0.64–0.86). The dashed diagonal line represents chance-level discrimination (AUC = 0.50). (**B**) Bootstrap AUC point estimates with 95% confidence intervals for all eight target genes in peripheral blood (blue circles) and buccal swab (red squares) samples. No gene reached the conventional threshold of AUC ≥ 0.90. *NR3C2* showed consistent moderate discriminative capacity across both matrices. The *MAOA* buccal swab estimate (AUC = 0.81) is based on 11 case samples and should be interpreted with caution.

ROC analysis was performed for each gene in blood and buccal swab samples (Figure 4A,B; Table 5). In buccal swabs, the highest AUC values were observed for *SLC6A3* (AUC = 0.80; 95% CI 0.69–0.89) and *NR3C1* (AUC = 0.72; 95% CI 0.57–0.85). In blood, the highest AUC was observed for *DRD2* (AUC = 0.73; 95% CI 0.63–0.81). *NR3C2* showed moderate AUC values in both matrices, with an AUC of 0.69 in blood (95% CI 0.59–0.79) and 0.76 in buccal swabs (95% CI 0.64–0.86). No individual gene reached an AUC of 0.90 or higher.

### 2.6. Correlations with Biochemical Parameters and Between-Group Biochemical Comparisons

Associations between gene expression levels and routine biochemical parameters were examined using Spearman’s rank correlation. A total of 576 correlations were tested in the case group and 416 in the control group; in both groups, the proportion of nominally significant correlations remained at chance level (5% and 6%, respectively), and no correlation survived FDR correction (Table 6). These findings indicate that the associations between gene expression and biochemical parameters should be regarded as exploratory and cannot be considered confirmatory. The failure of any correlation to exceed the FDR threshold also suggests that the gene expression findings represent a layer of biological information that is independent of routine metabolic status, which in turn supports the view that the observed transcriptional signals are not simply a byproduct of the biochemical profile [22,23].

Glucose and white blood cell count were higher in cases; urea, potassium, calcium, hemoglobin, hematocrit, and CRP were all lower (all q < 0.05; Table 6). CRP went the wrong way—down, not up—and has no clear explanation beyond acute presentation variability. Cortisol’s role in both changes is pharmacologically straightforward: it drives hepatic gluconeogenesis and redistributes leukocytes from the marginated pool into active circulation. Blood findings here are consistent with an acute stress response, not a surprise. None of it accounts for the gene expression differences, but it does provide information about the biological state of the case group at the time of sampling. The biochemical pattern and the *NR3C2* finding point in the same direction without one causing the other.

## 3. Discussion

The most consistent finding in this study was the downregulation of *NR3C2* in both peripheral blood and buccal swab samples from individuals who had made a suicide attempt. Two aspects of this finding are noteworthy. First, the effect held after FDR correction in both matrices independently. Second, no other gene in the panel came close to replicating across compartments—making *NR3C2* the only cross-matrix signal in the dataset.

*NR3C2* encodes the mineralocorticoid receptor (MR), which binds cortisol at approximately tenfold higher affinity than the glucocorticoid receptor and is primarily responsible for maintaining basal HPA-axis tone under non-stressed conditions [14,17]. The biology here is not trivial: impaired HPA feedback has been linked to suicide risk in multiple independent lines of work, including postmortem studies showing lower GR expression in the prefrontal cortex of individuals who died by suicide with a history of childhood adversity, and neuroendocrine studies documenting blunted cortisol suppression in suicide attempters [17]. This is also relevant because suicidal behavior has long been discussed within stress-related frameworks, particularly the stress–diathesis model, where individual vulnerability determines whether stress exposure translates into suicidal behavior [1,2]. The present findings do not prove altered central HPA-axis function, but they are compatible with the view that stress-axis-related transcriptional regulation might be detectable at the peripheral level. Because all samples were collected at a single acute presentation, the design cannot separate a pre-existing trait-level vulnerability from a state-dependent response to the attempt itself; the data are consistent with either interpretation.

The only prior study to directly examine *NR3C2* expression in suicide attempters reported under-expression specifically in individuals with a history of childhood trauma and high neglect scores but found no significant main effect in the overall case–control comparison [14]. The present study differs in two respects: it did not restrict the sample to patients with documented trauma histories, and it captures expression now of acute presentation rather than in a planned inpatient setting. That *NR3C2* downregulation emerged despite sample heterogeneity and without a trauma-stratified design may argue for a degree of robustness—though it equally underscores the need for replication in larger, better-characterized samples.

One question this finding does not settle is whether peripheral *NR3C2* mRNA reflects receptor density or function in the brain. It almost certainly does not do so in a simple, one-to-one fashion [22,23]. What it may reflect instead is a shared regulatory state—a systemic shift in how stress-responsive tissues are calibrating their glucocorticoid sensitivity at a given moment. That this shift appears across both blood and buccal epithelium, tissues with different cellular composition and embryonic origin, makes it harder to dismiss as a matrix-specific artifact. Outside the *NR3C2* finding, blood and buccal swabs told largely different stories. *SLC6A4* and *DRD2* reached significance in blood only; *SLC6A3*, *NR3C1*, *GABRA2*, and *GABBR2* did so only in swabs.

Blood *SLC6A4* levels were lower in cases. Consoloni et al. found something similar—in patients with major depressive disorder, lower peripheral *SLC6A4* mRNA early in follow-up predicted a deteriorating course of suicidal ideation [6]. Genetic variants and mRNA levels are not interchangeable measures: one reflects what a person inherited, the other reflects what their tissue is doing at a given moment. This distinction is part of the rationale for treating expression data as a complement to genotyping rather than a substitute for it. The *SLC6A4* result is directionally consistent with the serotonergic literature, but it does not stand alone.

*DRD2* moved in the opposite direction—higher in cases. The dopamine literature on suicidal behavior generally points toward underactivity: diminished reward processing, impaired inhibitory control, impulsivity linked to reduced striatal D2 signaling [1,10]. A rise in peripheral mRNA does not map onto that cleanly. The likely explanation is that mRNA levels and receptor function are not the same thing—a transcript increase can represent a compensatory shift rather than a direct index of activity [10,13]. Yamamoto et al. noted this problem specifically for peripheral *DRD2* data [13]. The blood finding stands, but its mechanistic meaning cannot be determined from this design.

The swab data diverged substantially from the blood data. Every gene that cleared FDR in buccal swabs moved in the same direction: down in cases. *NR3C2*, *NR3C1*, *SLC6A3*, *MAOA*, *GABRA2*, and *GABBR2* were all reduced, spanning two distinct receptor systems, which argues against this pattern being attributable to chance alone. Postmortem prefrontal tissue from suicide decedents has shown epigenetic silencing of GABAergic genes, interpreted as impaired inhibitory regulation at the cortical level [15,18]. Prolonged glucocorticoid exposure under chronic stress can also suppress GABAergic receptor transcription, tying inhibitory signaling directly to HPA-axis state [26,27]. Buccal epithelium is neuroectodermal—embryologically closer to neural tissue than the mesodermal-derived cells that make up most of what blood contains. This developmental distinction may partly explain why the swab profile diverges from that of blood. The joint downregulation of *NR3C2* and GABAergic genes in swabs is not incidental: mineralocorticoid receptor signaling regulates GABAergic interneuron activity, and reduced MR tone under chronic stress has been linked to prefrontal inhibitory deficits [17,19]. Whether that relationship extends to the peripheral transcriptional level is still an open question, but the directional pattern is consistent with it.

*MAOA* showed the largest effect size in buccal swabs, but the comparison rested on eleven case samples. This limits the stability of the estimate and increases the likelihood of an inflated effect size. *MAOA* is biologically relevant—it is involved in the degradation of serotonin and dopamine, and has been linked to impulsivity and suicidal behavior, particularly in the context of early-life stress [8,9]. Even so, this finding should be kept at the level of a hypothesis, not used to carry the main argument. *GABBR2* moved in opposite directions across matrices—upregulated in blood, downregulated in swabs—and both reached FDR significance. Rather than a contradictory result, this divergence is consistent with tissue-specific regulation: if *GABBR2* expression were driven by a single, system-wide biological state, it would be expected to move in the same direction in both matrices. Blood-derived immune cells and buccal epithelial cells are exposed to different local signaling environments and express different complements of regulatory machinery [26,27], which may account for the opposite-direction finding. This has a practical implication for future peripheral expression studies of suicidal behavior: the matrix selected can shape which signals are detected.

Gene expression and routine biochemistry did not correlate after FDR correction in either group; if these expression differences were driven by concurrent metabolic disruption, a correlation with glucose, electrolytes, or CRP would have been expected, and none was observed. The transcriptional signal and the biochemical picture therefore appear to carry separate information [22,23], although they still converge directionally: raised glucose and leukocytes alongside lower urea, potassium, calcium, hemoglobin, and hematocrit sketch the same cortisol-driven acute state that *NR3C2* downregulation points toward.

No gene cleared AUC ≥ 0.90. That threshold marks the lower bound for clinical decision support, and none of the eight genes came close. This was not unexpected—suicidal behavior does not reduce to a single peripheral transcript. The AUC values describe where each gene sits on the discriminative spectrum; they do not justify clinical use. Whether a combination of these genes with cortisol measures, psychometric scores, or clinical risk indices would perform better is a question this study cannot answer.

### 3.1. Limitations

Cases were 71% female; controls were 43% female, a difference with direct interpretive consequences. *MAOA* sits on the X chromosome, and its expression differs by sex—the swab finding cannot be cleanly separated from the sex distribution without controlling for it [23]. The broader monoaminergic findings carry the same problem: sex is a known modifier of monoaminergic transcription across tissues [23], and this dataset cannot account for that. A subject-level sensitivity analysis adjusting for these factors, and a comparison of included versus excluded buccal cases to assess non-random missingness, were both attempted but could not be reliably constructed, as the available data sources do not preserve a consistent subject-level identifier linking gene-expression records to sociodemographic data; this is noted here as an open limitation rather than resolved.

A stratified analysis by method of attempt was not undertaken: 63 of 69 cases (91.3%) presented after drug or substance ingestion, and each remaining method accounted for only one or two cases, entirely confounded with sex; the resulting cell sizes leave no meaningful statistical power for this comparison. Substance and alcohol use was recorded only in cases. Psychiatric medication use was similarly restricted: 40.6% of cases reported current psychotropic medication, compared with 1.4% of controls. Both substance exposure and psychotropic medication are known to influence monoaminergic and serotonergic gene expression, and their effects cannot be separated from the biological state associated with the attempt itself in this design. The design is cross-sectional, which means there is no way to tell whether these expression differences existed before the event, emerged in response to it, or reflect something else happening at admission. Separating trait from state would require repeated measures—something this study was not built to do. And with complete eight-gene data available in only eleven individuals, multivariable analysis was not feasible.

A few things worked in the study’s favor. Both matrices came from the same individuals at the same time—no cohort splitting, no gap between collections. Without that paired structure, matrix-specific differences would be uninterpretable. The gene panel had a defined biological rationale rather than being assembled opportunistically. FDR correction was applied consistently, and ROC analysis was included to give a direct read on discriminative capacity. The central finding was not one gene but a pattern: one signal that replicated across matrices, and several others that were entirely matrix dependent.

### 3.2. Scope and Interpretation

Clinical biomarker development was not the aim here. No gene cleared a threshold that would support clinical use, and the cross-sectional design makes causal claims impossible. What the data establish is narrower: acute suicide attempt presentation is accompanied by measurable transcriptional differences in two separate peripheral matrices, those differences organize around biologically coherent systems, and *NR3C2* is the only gene that showed up consistently in both. Cross-matrix concordance and diagnostic validity are separate claims, and only the first is established here: agreement between blood and buccal swab expression indicates that the *NR3C2* signal is not an artifact of one tissue’s handling or composition, but it does not by itself demonstrate that this signal discriminates suicide attempt from non-attempt status with clinical accuracy. That latter question was tested directly through ROC analysis, where discriminative performance remained moderate.

Blood and buccal swabs are picking up different signals. Using one as a stand-in for the other would discard information—the data here make that concrete rather than theoretical. What comes next is replication: larger samples, clinical stratification by sex and substance use, longitudinal collection, and validation at the protein and epigenetic level. This study defines the question more precisely than it answers it. The confounder-adjustment constraint noted in Section 3.1 applies directly here: without it, the associations reported should be read as hypothesis-generating candidates rather than confounder-independent signals.

## 4. Materials and Methods

### 4.1. Study Design, Setting, and Case–Control

This case–control, cross-sectional study was conducted between November 2024 and November 2025 at the Emergency Department of Bakırköy Dr. Sadi Konuk Training and Research Hospital, with laboratory analyses carried out at the Department of Molecular Medicine, Aziz Sancar Institute of Experimental Medicine, Istanbul University, and at the Faculty of Pharmacy research laboratories, Acibadem Mehmet Ali Aydınlar University. The protocol was approved by the Clinical Research Ethics Committee of Acibadem Mehmet Ali Aydınlar University (ATADEK-2024-19/9) on 12 December 2024, and the study followed the Declaration of Helsinki throughout.

Informed consent was obtained directly from participants where possible; where consciousness level did not allow this, a first-degree relative or the attending emergency team completed the process on the patient’s behalf. Cases were adults aged 18 years or older presenting after a suicide attempt, without an additional physician-assigned self-harm diagnosis as a separate entity. Controls were adults of the same age range presenting to the same department for unrelated reasons, with no suicide attempt history and no current ideation. Minors, patients presenting for reasons that did not meet either group’s criteria, or completed-suicide cases fell outside the study’s scope. A priori power analysis, based on an assumed effect size of d = 0.52 drawn from comparable studies, indicated a minimum of 59 participants per group (two-sided α = 0.05, power = 0.80). Given the psychological vulnerability of this population, the ethics committee approved recruitment up to 100 individuals per group; consecutive enrollment over the study period yielded 69 cases and 72 controls.

### 4.2. Sample Collection and Clinical Data

A structured Emergency Department Suicide Attempt Registration Form captured sociodemographic and clinical variables at presentation: age, sex, place of birth, marital status, employment, method and frequency of attempt, psychiatric diagnosis history, current medication, and alcohol, tobacco, or substance use. Biochemical parameters ordered by the attending physician as part of routine care—glucose, urea, uric acid, C-reactive protein, total bilirubin, AST, ALT, GGT, calcium, sodium, potassium, chloride, albumin, white blood cell count, hemoglobin, hematocrit, lymphocytes, and eosinophils—were pulled from hospital records rather than measured separately for this study.

Blood was drawn into sterile 10 mL EDTA tubes; buccal mucosa was sampled with sterile swab applicators. Both sample types were collected from each participant at the same visit and kept at +4 °C until RNA isolation began.

### 4.3. RNA Isolation from Blood and Buccal Swab Samples

**Figure 5:**
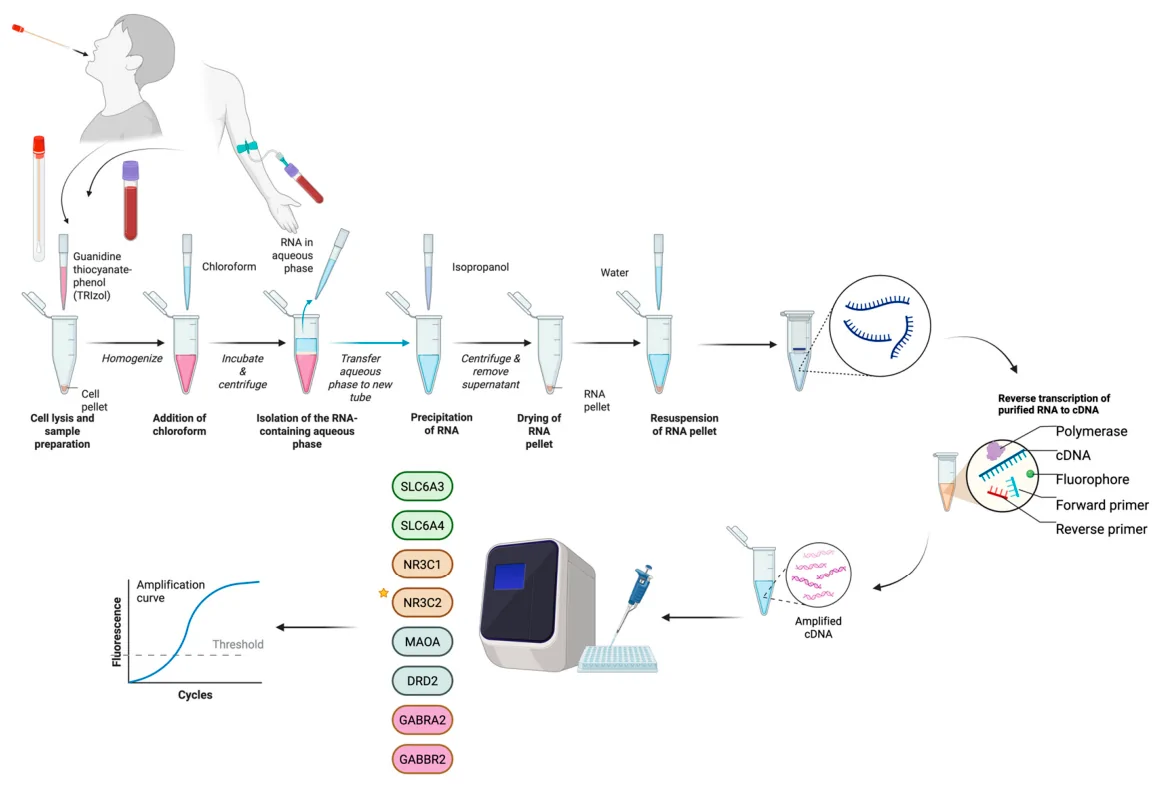
Schematic workflow of RNA isolation, cDNA synthesis, and qRT-PCR gene expression analysis in blood and buccal swab samples. Created in BioRender. Bireller, S. (2026) (https://BioRender.com/mt0fbvq, (accessed on 4 July 2026)).

Total RNA was isolated from both matrices using a guanidinium thiocyanate–phenol (TRIzol)-based extraction, adapted separately for whole blood and for buccal epithelial cells collected on swabs (Figure 5).

Whole blood (500 µL) was lysed in 800 µL TRIzol reagent by brief high-speed vortexing and a 5 min incubation, with all steps carried out on dry ice to limit foaming and sample warming. The aqueous phase was separated by adding 200 µL chloroform, vortexing for 15–20 s, and centrifuging at 15,000× *g* (4 °C, 15 min) after a 2–3 min incubation. Five hundred microliters of the resulting supernatant were transferred to a fresh tube and re-extracted with a second 200 µL aliquot of chloroform (18,000× *g*, 10 min) to reduce genomic DNA carryover, yielding 400 µL of clarified aqueous phase. RNA was precipitated with 400 µL isopropanol—equivalent to 0.5 volumes of the TRIzol used for lysis, per the manufacturer’s standard ratio—at −40 °C for 18–24 h, followed by centrifugation at 18,000× *g* (4 °C, 10 min). The pellet was washed twice in 75% ethanol (1 mL, then 500 µL; 18,000× *g* for 5 min and 20,627× *g* for 3 min), dried briefly on ice, and resuspended in 40 µL RNase-free water with a 10 min, 60 °C incubation.

Buccal epithelial cells were recovered by transferring swab heads into 1 mL PBS with sterile forceps, vortexing for 30 s, and pelleting at 12,000× *g* (4 °C, 5 min); the resulting pellet was lysed in 750 µL TRIzol for 5 min. Aqueous-phase separation used the same chloroform step described above (200 µL, 12,000× *g* for 15 min), giving 400 µL of supernatant. Isopropanol was again added at 0.5 volumes of the TRIzol used (375 µL), with precipitation, washing (12,000× *g*, 5 min), and resuspension (20 µL RNase-free water, 60 °C, 10 min) otherwise following the blood protocol, except that only a single ethanol wash was performed.

All steps were carried out under RNase-free conditions in a laminar flow cabinet, and isolated RNA was stored at −80 °C pending downstream analysis.

### 4.4. RNA Quantification and Quality Assessment

Concentration and purity were read on a NanoDrop (Maestrogen Inc., Hukou Township, Taiwan) at 260, 230, and 280 nm, blanked against RNase-free water. Samples were carried forward only if the OD260/OD230 ratio sat near 2.0. Buccal swab samples were disproportionately affected by pre-analytical variability in collection and storage, which reduced RNA yield and quality more often than in blood; consequently, a larger number of buccal samples—particularly among cases—did not meet the quality threshold and were excluded from downstream analysis. As a result, the number of samples contributing to each gene comparison varies across the panel and is reported individually in the corresponding results tables.

### 4.5. cDNA Synthesis

Reverse transcription used the OneScript^®^ Plus cDNA Synthesis Kit (Applied Biological Materials Inc., Vancouver, BC, Canada): 4 µL 5× RT buffer, 1 µL dNTP mix, 1 µL random primer, 8 µL nuclease-free water, 1 µL enzyme, and 5 µL of concentration-normalized RNA template, assembled on ice to a 20 µL final volume. Cycling ran on a BIO-RAD T100 (Bio-Rad Laboratories, Hercules, CA, USA)—25 °C for 10 min, 55 °C for 30 min, 85 °C for 5 min, then a 4 °C hold. cDNA quality was checked by NanoDrop before storing leftover RNA at −80 °C.

### 4.6. qRT-PCR Gene Expression Analysis

Gene expression was quantified from the synthesized cDNA using BlasTaq 2X qPCR Master Mix (Applied Biological Materials Inc., Vancouver, BC, Canada) with the primer pairs listed in Table S1, targeting the eight genes of interest and *GAPDH* as the endogenous reference. Reactions were assembled in 96-well plates, with 18 µL of a primer-specific master mix (0.25 µL each of forward and reverse primer, 5 µL of 2× BlasTaq master mix, and nuclease-free water to volume) dispensed per well, followed by 2 µL of cDNA template, for a final reaction volume of 20 µL. Each sample was run in technical triplicate for every gene, and the mean Ct value across replicates was used for downstream analysis. One no-template control well was included for each primer pair, containing master mix only.

Amplification was performed on a LightCycler^®^ 96 (Roche Diagnostics GmbH, Mannheim, Germany) using a two-step cycling protocol: an initial pre-incubation at 95 °C for 150 s (one cycle) for polymerase activation and template denaturation, followed by 45 cycles of denaturation at 95 °C for 15 s and combined annealing/extension at 52–56 °C (primer-dependent) for 10 s. Cycling concluded with a melt-curve step at 95 °C for 45 s and a final cooling step at 37 °C for 30 s. Cycle threshold (Ct) values were exported from the instrument software and normalized to *GAPDH* to derive ΔCt values for each gene and sample, which served as the basis for the statistical analyses described in Section 4.7.

### 4.7. Statistical Analyses

Prior to between-group comparisons, the normality of ΔCt values for each gene was assessed using the Shapiro–Wilk test. Where the assumption of normality was met, Welch’s *t*-test was applied; otherwise, the Mann–Whitney U test was used. With eight genes tested per matrix, uncorrected *p* values were adjusted for multiple comparisons using the Benjamini–Hochberg procedure; the resulting FDR-corrected q values served as the primary criterion for significance, with a threshold of q < 0.05. Effect sizes were quantified with Hedges’ g; bootstrap resampling was used to derive 95% confidence intervals. Group differences in gene expression are also reported as log_2_ fold change (log_2_FC): values above zero reflect higher expression in cases, values below zero reflect lower expression. For Hedges’ g, a positive value reflects a higher ΔCt in cases, corresponding to downregulated expression; a negative value reflects a lower ΔCt in cases, corresponding to upregulated expression. The discriminative capacity of individual genes was evaluated by receiver operating characteristic (ROC) curve analysis; area under the curve (AUC) values and their 95% confidence intervals were estimated by bootstrap resampling. Associations between gene expression levels and routine biochemical parameters were examined using Spearman’s rank correlation coefficient; resulting correlation coefficients were also subjected to Benjamini–Hochberg FDR correction. A total of 576 correlations were tested in the case group (eight genes × 18 biochemical parameters) and 416 in the control group. Between-group comparisons of biochemical parameters were performed separately across the 11 parameters available in both groups and were likewise corrected for multiple comparisons using the Benjamini–Hochberg method. All analyses were performed using GraphPad Prism (version 10) and Microsoft Excel.

## 5. Conclusions

Adults presenting after a suicide attempt showed peripheral gene expression differences across the neurobiological systems targeted by this panel. Of the eight genes examined, only *NR3C2* was consistently downregulated in both blood and buccal swabs—the one signal that did not depend on which tissue was sampled. Other findings were matrix-specific, and *GABBR2* changed in opposite directions across the two sample types. Taken together, these results indicate that blood and buccal swabs capture partially distinct biological information and should not be treated as interchangeable in peripheral transcriptomic studies of suicidal behavior. The data do not support a single-gene biomarker interpretation. Paired peripheral sampling is feasible in acute clinical settings and captures biological information that single-matrix designs would miss. The data are better read as framing a research question than answering one: what combination of peripheral transcriptomics, neuroendocrine profiling, and clinical characterization would have the power to resolve the vulnerability that makes the difference between distress and suicidal behavior?

## Figures and Tables

**Table 1:** Sociodemographic and clinical characteristics of cases and controls.

Variable	Cases (*n* = 69)	Controls (*n* = 72)	*p*
Demographic characteristics			
Age (mean ± SD)	33.25 ± 14.06	35.15 ± 14.95	0.439
Sex, *n* (%)			
Female	49 (71.0%)	31 (43.1%)	0.003
Male	19 (27.5%)	40 (55.6%)	
Unknown	1 (1.4%)	1 (1.4%)	
Marital status, *n* (%)			
Married	27 (39.1%)	30 (41.6%)	>0.05
Single	40 (58.0%)	42 (58.4%)	
Clinical characteristics			
Psychiatric diagnosis history, *n* (%)	41 (59.4%)	1 (1.4%)	<0.001
Reported psychiatric medication use, *n* (%)	28 (40.6%)	1 (1.4%)	<0.001
Substance/alcohol use, *n* (%)	Present	Absent	0.019
Repeat attempt history, *n* (%)			
First attempt	44 (63.8%)	—	
Second attempt	11 (15.9%)	—	
Third attempt	6 (8.7%)	—	
Fourth attempt	2 (2.9%)	—	
Unknown	6 (8.7%)	—	
Method of suicide attempt (cases only)			
Drug/substance ingestion	63 (91.3%)	—	—
Sharp/cutting instrument	2 (2.9%)	—	—
Hanging	2 (2.9%)	—	—
Jumping from height	1 (1.4%)	—	—
Unknown	1 (1.4%)	—	—

Abbreviations: SD, standard deviation. Age was compared using the Mann–Whitney U test; all categorical variables were compared using Pearson’s chi-square test. All *p* values are two-tailed; statistical significance was set at *p* < 0.05. The p value for sex reflects the female/male comparison (unknown category excluded). Repeat attempt history and method data apply to cases only; these variables were not applicable in controls (—).

**Table 2:** Gene expression in blood samples: case–control comparison.

Gene	*n* (Cases)	*n* (Controls)	Median ΔCt Cases	Median ΔCt Controls	log_2_FC	q (FDR)	Hedges g (95% CI)	Direction
*SLC6A3*	58	61	5.40	3.06	−2.34	0.3152	+0.04 (−0.31; +0.42)	—
*SLC6A4*	62	67	4.99	2.02	−2.97	0.0007	+0.60 (+0.24; +1.05)	↓
*NR3C1*	60	60	2.09	0.46	−1.63	0.2886	+0.15 (−0.22; +0.52)	—
*NR3C2*	57	63	4.10	−0.49	−4.59	0.0007	+0.82 (+0.45; +1.21)	↓
*DRD2*	64	63	0.79	3.33	+2.54	0.0001	−0.98 (−1.28; −0.69)	↑
*MAOA*	56	57	5.85	2.95	−2.90	0.1286	+0.28 (−0.08; +0.67)	—
*GABRA2*	55	54	3.26	2.86	−0.40	0.5425	+0.05 (−0.34; +0.43)	—
*GABBR2*	65	65	1.21	2.11	+0.90	0.0317	−0.56 (−0.91; −0.25)	**↑**

Abbreviations: *n* (Cases), number of cases; *n* (Controls), number of controls; ΔCt, cycle threshold normalized to GAPDH (higher ΔCt = lower expression); log_2_FC, log_2_ fold change—negative values indicate downregulation in cases, positive values upregulation; q, FDR-corrected *p* value by Benjamini–Hochberg across 8 genes per matrix (q < 0.05 significant); Hedges g, effect size with bootstrap 95% CI—positive g: higher ΔCt in cases (downregulation); negative g: lower ΔCt in cases (upregulation); Direction, expression direction in cases.

**Table 3:** Gene expression in buccal swab samples: case–control comparison.

Gene	*n* (Cases)	*n* (Controls)	Median ΔCt Cases	Median ΔCt Controls	log_2_FC	q (FDR)	Hedges g (95% CI)	Direction
*SLC6A4*	37	57	4.95	4.33	−0.62	0.3369	+0.22 (−0.22; +0.69)	—
*NR3C1*	19	47	6.60	3.91	−2.69	0.0077	+0.86 (+0.31; +1.53)	↓
*SLC6A3*	34	64	5.32	2.79	−2.54	<0.0001	+0.93 (+0.52; +1.36)	↓
*NR3C2*	31	54	10.39	5.48	−4.91	0.0004	+0.94 (+0.48; +1.47)	↓
*DRD2*	34	69	−0.49	0.26	+0.75	0.2197	−0.28 (−0.70; +0.16)	—
*MAOA*	11	69	9.32	4.22	−5.10	0.0030	+1.26 (+0.32; +2.43)	↓
*GABRA2*	29	51	8.07	5.33	−2.74	0.0077	+0.69 (+0.22; +1.23)	↓
*GABBR2*	28	63	4.32	2.50	−1.82	0.0077	+0.51 (−0.04; +1.17)	↓

Abbreviations: as in Table 2; *n*(Cases), number of cases; *n*(Controls). The MAOA comparison in buccal swabs is based on only 11 case samples; the bootstrap confidence interval is correspondingly wide, and this finding should be interpreted with caution. All genes reaching FDR significance in swabs showed a consistent direction of downregulation (↓).

**Table 4:** Cross-matrix gene expression profile: blood versus buccal swab.

Gene	Blood q (FDR)	Blood Direction	Swab q (FDR)	Swab Direction	Cross-Matrix Consistency	Note
*SLC6A3*	0.3152	—	<0.0001	↓	Swab-specific	Not significant in blood
*SLC6A4*	0.0007	↓	0.3369	—	Blood-specific	Not significant in swab
*NR3C1*	0.2886	—	0.0077	↓	Swab-specific	Not significant in blood
*NR3C2*	0.0007	↓	0.0004	↓	Both matrices	Only consistent gene across both matrices
*DRD2*	0.0001	↑	0.2197	—	Blood-specific	Not significant in swab
*MAOA*	0.1286	—	0.0030†	↓	Swab-specific	n(cases) = 11; wide CI, interpret with caution
*GABRA2*	0.5425	—	0.0077	↓	Swab-specific	Not significant in blood
*GABBR2*	0.0317	↑	0.0077	↓	Opposite direction	↑ in blood, ↓ in swab

Abbreviations: q: FDR-corrected *p* value by Benjamini–Hochberg (q < 0.05 significant). NR3C2 alone was downregulated in both matrices (↓). GABBR2 moved in opposite directions—up in blood, down in swabs. The MAOA swab result rests on 11 case samples and should be read accordingly.

**Table 5:** Discriminative performance of individual genes: AUC and 95% bootstrap confidence intervals.

Gene	AUC (Blood)	95% CI Lower	95% CI Upper	AUC (Swab)	95% CI Lower	95% CI Upper
HPA axis						
*NR3C1*	0.57	0.50	0.67	0.72	0.57	0.85
*NR3C2*	0.69	0.59	0.79	0.76	0.64	0.86
Serotonergic/Dopaminergic axis						
*SLC6A3*	0.56	0.50	0.67	0.80	0.69	0.89
*SLC6A4*	0.69	0.60	0.78	0.56	0.50	0.67
*DRD2*	0.73	0.63	0.81	0.56	0.50	0.67
*MAOA*	0.60	0.51	0.71	0.81	0.62	0.95
GABAergic axis						
*GABRA2*	0.53	0.50	0.65	0.71	0.58	0.82
*GABBR2*	0.62	0.53	0.72	0.68	0.54	0.82

Abbreviations: AUC: area under the receiver operating characteristic curve. 95% CI: bootstrap confidence interval. No gene reached the conventional clinical threshold of AUC ≥ 0.90. NR3C2 showed consistent discriminative performance across both matrices. MAOA AUC in swabs is based on 11 case samples; the confidence interval is wide.

**Table 6:** Comparison of routine biochemical parameters between cases and controls.

Parameter	n (Cases)	n (Control)	Median Cases	Median Controls	Raw *p*	q (FDR)	Direction
Parameters significant after FDR correction (q < 0.05)							
Glucose (GLU), mg/dL	68	72	102.0	91.0	0.003	0.005	↑
White blood cell count (WBC), ×10^3^/µL	68	72	8.60	7.33	<0.001	<0.001	↑
Urea (URE), mg/dL	68	72	19.0	25.5	<0.001	<0.001	↓
Potassium (K), mEq/L	68	72	4.00	4.42	<0.001	<0.001	↓
Calcium (Ca), mg/dL	68	72	9.21	9.50	<0.001	<0.001	↓
Hemoglobin (Hb), g/dL	68	72	12.5	14.0	<0.001	<0.001	↓
Hematocrit (HCT), %	68	72	38.6	42.5	<0.001	<0.001	↓
CRP, mg/L	68	72	0.70	0.83	0.008	0.010	↓
Parameters not significant after FDR correction (q ≥ 0.05)							
Sodium (Na), mEq/L	68	72	139.0	139.0	0.965	0.965	—
AST, U/L	68	72	17.6	16.5	0.314	0.346	—
ALT, U/L	68	72	12.5	14.0	0.292	0.346	—
Parameters not measured in controls *							
Uric acid (UA), mg/dL	68	—	4.00	—	—	—	—
Total bilirubin (T.BIL), mg/dL	68	—	0.40	—	—	—	—
GGT, U/L	68	—	10.0	—	—	—	—
Albumin (ALB), g/L	68	—	43.0	—	—	—	—
Chloride (Cl), mEq/L	68	—	105.0	—	—	—	—

Abbreviations: as in Table 2; *n* (Cases), number of cases; *n* (Controls). Values are medians. All comparisons used the Mann–Whitney U test; raw *p* values were corrected by Benjamini–Hochberg across 11 parameters (q < 0.05 threshold). CRP ran lower in cases—the opposite of what acute stress would predict. Individual variability at the time of emergency presentation is the most plausible explanation; treat with caution. Albumin values are in g/L (reference range 35–50 g/L). * These parameters were not available in controls. No comparison was run. LYM and EOS were excluded—the unit problem across samples made any comparison uninterpretable.

## Data Availability

The data presented in this study are available on request from the corresponding author; they are not publicly available due to ethical restrictions related to participant privacy.

## Supplementary Materials

The following supporting information can be downloaded at: https://www.mdpi.com/article/10.3390/ijms27167185/s1.

## Abbreviations

The following abbreviations are used in this manuscript:

*DRD2*	Dopamine Receptor D2
FDR	False Discovery Rate
*GABRA2*	Gamma-Aminobutyric Acid Type A Receptor Subunit Alpha-2
*GABBR2*	Gamma-Aminobutyric Acid Type B Receptor Subunit 2
*GAPDH*	Glyceraldehyde-3-Phosphate Dehydrogenase
HPA	Hypothalamic–Pituitary–Adrenal (Axis)
*MAOA*	Monoamine Oxidase A
mRNA	Messenger Ribonucleic Acid
*NR3C1*	Nuclear Receptor Subfamily 3 Group C Member 1
*NR3C2*	Nuclear Receptor Subfamily 3 Group C Member 2
RNA	Ribonucleic Acid
*SLC6A3*	Solute Carrier Family 6 Member 3
*SLC6A4*	Solute Carrier Family 6 Member 4
qRT-PCR	Quantitative Reverse Transcription Polymerase Chain Reaction
