# Supplementary material for: A Cross-Matrix Peripheral Transcriptional Signature in Suicide Attempt: NR3C2 Downregulation in Blood and Buccal Swab Samples
Source: Int J Mol Sci. 2026 Aug 11;27(16):7185. doi: 10.3390/ijms27167185 (PMC13513759; doi:10.3390/ijms27167185)
Supplement: Supplementary file 1 [file ijms-27-07185-s001.zip › ijms-4448650-supplementary.pdf]

Supplementary methodological details, including gene-specific primer sequences and melting temperatures used for RT-qPCR analysis, are summarized in Table S1.

Table S1. Gene-specific primer sequences and melting temperatures

Genes	Sequence (F)	Sequence (R)	Tm (°C)
SLC6A3	AGCCTACATCTTCCCCGACT	CCCGTCCAAATACCCACCTC	52
SLC6A4	GGGATTGACACGTCGGGATT	GGACTGCCATGTAGCAAATAGG	52
NR3C1	TTCATCAGAGCACACCAGGC	TGTTCTTCCCCTTCTTTAGCCC	52
NR3C2	ATAGAGGAGGAGATGGTCAGC	TGTATAAATGGTGGGCAGGCA	52
DRD2	GCCTTCTGCCACTCTTTTGC	CTGAAGTGTGTGGTGCATGG	52
MAOA	GTGTGTGCTTTGCCACTGAA	ATGCCTCATTGTGACCTCCTG	53
GABRA2	GCATAAGCCACTTTGGGGAGA	CCCATGCTGTCCTGCTAGT	52
GABBR2	TGAGAGCCCTTTTCCCTCCT	AACAGAAGGCAGTCAACGCT	52
GAPDH	TTCTTTTGCGTCGCCAGCC	TCCCGTTCTCAGCCTTGAC	56
